# Sarcopenia is a predictive factor for intestinal resection in admitted patients with Crohn’s disease

**DOI:** 10.1371/journal.pone.0180036

**Published:** 2017-06-23

**Authors:** Shigeki Bamba, Masaya Sasaki, Azusa Takaoka, Kenichiro Takahashi, Hirotsugu Imaeda, Atsushi Nishida, Osamu Inatomi, Mitsushige Sugimoto, Akira Andoh

**Affiliations:** 1 Department of Medicine, Shiga University of Medical Science, Otsu, Shiga, Japan; 2 Division of Clinical Nutrition, Shiga University of Medical Science, Otsu, Shiga, Japan; Kurume University School of Medicine, JAPAN

## Abstract

The relationship between skeletal muscle volume and the prognosis of patients with inflammatory bowel disease (IBD) remains undetermined. We conducted a retrospective study of 72 IBD patients who were admitted to the hospital due to disease exacerbation. We enrolled IBD patients who had undergone abdominal computed tomography and assessed the nutritional indices, such as the Onodera’s prognostic nutritional index (O-PNI) and the controlling nutritional status (CONUT) index. The L3 skeletal muscle index (SMI), which is the ratio of the cross-sectional area of skeletal muscles at the level of the third lumbar (L3) vertebra to the height squared, was used to identify sarcopenia. Sarcopenia, defined as a low SMI, was observed in 42% of all IBD patients (37% with Crohn’s disease (CD) and 48% with ulcerative colitis (UC)). In UC patients, the O-PNI and CONUT values, height, and albumin levels were significantly lower than in CD patients. The SMI strongly correlated with sex, body weight, albumin level, and O-PNI in IBD patients. Multivariate analysis using the Cox regression model demonstrated that the presence of sarcopenia (P = 0.015) and disease type (CD or UC) (P = 0.007) were significant factors predicting intestinal resection. The cumulative operation-free survival rate was significantly lower for sarcopenic patients than in all IBD patients (P = 0.003) and a stratified analysis of CD patients (P = 0.001) using the Kaplan–Meier method and log-rank test. The L3 skeletal muscle area is a prognostic factor for intestinal resection in patients with CD.

## Introduction

Inflammatory bowel diseases (IBD), such as ulcerative colitis (UC) and Crohn’s disease (CD), are chronic gastrointestinal diseases that are associated with protein-energy malnutrition (PEM) [1, 2]. PEM is caused by low dietary intake, enhanced energy expenditure due to inflammation, impaired digestion and absorption, and protein leakage from ulcerative lesions [2–6]; it leads to decreases in skeletal muscle and adipose tissue volumes. Altered body composition, such as reduced fat-free mass, has been reported in IBD patients [7, 8]. Additionally, decreased skeletal muscle volume has been observed in 60% of CD patients in clinical remission [9]. A recent report demonstrated decreased phosphorylated:total Akt ratio in muscle biopsy obtained from CD patients, suggesting impaired activation of muscle protein synthesis pathways, notably the IGF-1-Akt pathway [10]. The involvement of upstream proinflammatory cytokines, such as interleukin-6 or tumor necrosis factor-α, was suspected.

There are several reports concerning altered body composition and clinical outcomes of IBD. Zhang et al. reported the relationships between decreased skeletal muscle volume and postoperative complications [11]. Furthermore, Holt et al. reported that visceral adiposity but not low skeletal muscle predicted endoscopic recurrence after surgery in CD [12]. However, the relationship between reductions in skeletal muscle volume and prognoses of IBD patients has yet to be elucidated.

The description of sarcopenia was first made in 1989 by Rosenberg to describe changes in body composition and related functions, such as age-related loss of muscle mass and function [13]. Sarcopenia can be considered primary (or age-related) when no other cause is evident except for aging itself or as secondary when causes apart from aging are evident [14, 15]. Sarcopenia is associated with age, activity, disease and nutritional statuses [14]. Recently, an association between disease status and prognosis was reported in the field of malignant neoplasms and chronic diseases, such as heart failure, obstructive pulmonary disease, diabetes mellitus, kidney disease, connective tissue disease, tuberculosis, and other wasting conditions [16–23]. Recently, there has been increased interest in the clinical importance of secondary sarcopenia [15].

According to the European Working Group on Sarcopenia in Older People (EWGSOP), the definition of sarcopenia is low muscle mass and low muscle strength or low physical performance [14]. Skeletal muscle volume is reported to have a strong correlation with physical performance, such as gait speed, as well as with muscle strength, such as grip strength or hip flexion force [24, 25]. Furthermore, the cross-sectional area of skeletal muscles at the level of the third lumbar (L3) vertebra is considered a surrogate marker of total skeletal muscle volume [26]. The L3 skeletal muscle index (SMI), which is the L3 skeletal muscle area divided by the height squared, is used to assess skeletal muscle volume [16, 18, 20]. Herein, we performed a retrospective study to investigate the relationship between the SMI and the prognosis of IBD.

## Materials and methods

### Study population and clinical data

This retrospective study was conducted with the approval of the Ethics Committee of the Shiga University of Medial Science (28–058). The Ethics Committee of the Shiga University of Medial Science waived the need for consent from individuals. The opt-out information was published in the following URL (http://www.shiga-med.ac.jp/hospital/doc/department/department/digestive_int/index.html). The clinical data was collected from medical records and the data was anonymized for analysis. The subjects were a consecutive series of IBD patients admitted to the Shiga University of Medical Science Hospital due to disease exacerbation and were assessed by the nutrition support team (NST) of the Shiga University of Medical Science Hospital between January 2011 and December 2016. A total 72 IBD patients had undergone abdominal computed tomography (CT) two weeks before or after the admission; they had also been assessed by the NST for their nutritional status and resting energy expenditure (REE) using indirect calorimetry within one week after admission. The REE and respiratory quotient (RQ) were measured via a computed open-circuit IC (AE-300S, Minato Medical Science Corporation, Osaka, Japan), as previously described [5, 6, 27–30]. The basal energy expenditure (BEE) was calculated using the Harris-Benedict equation [31]. For nutritional assessments, the subjective global assessment (SGA) [32], controlling nutritional status (CONUT) [33], malnutrition universal screening tool (MUST) [34], nutritional risk screening 2002 (NRS-2002) [35], and Onodera’s prognostic nutritional index (O-PNI) [36, 37] were used. O-PNI was calculated based on the serum albumin level and total lymphocyte count, using the following equation: O-PNI = 10 × [serum albumin (g/dL) + 0.005 × total lymphocyte count (/mL)]. Assessments of nutritional status and REE were performed after admission. Clinical backgrounds, laboratory data, clinical symptoms, endoscopic findings, and other clinical data related to intestinal resection, were collected retrospectively.

### Assessment of muscle volume and definition of sarcopenia

The cross-sectional area of skeletal muscles at the level of the L3 vertebra was measured on CT images using image analysis software (OsiriX MD version 7.5.1, Pixmeo, Geneva, Switzerland), which enabled the identification of a specific tissue demarcation by the Hounsfield unit from -29 to +150 [38]. The skeletal muscles at the L3 level include the psoas, erector spinae, quadratus lumborum, transversus abdominis, internal oblique, external oblique, and rectus abdominis. The L3 skeletal muscle index (SMI) which is the L3 skeletal muscle area divided by the height squared is used to assess the skeletal muscle volume. The cut-off values of the SMI for identifying sarcopenia in liver disease are 42 cm^2^/m^2^ for men and 38 cm^2^/m^2^ for women [39]. Patients with an SMI below the cut-off value were defined as sarcopenia.

### Statistical analyses

All statistical analyses were performed using Prism, version 6.05 (GraphPad, San Diego, CA), and IBM SPSS Statics, version 20.0 (IBM Corporation, Armonk, NY, USA). The χ2–test and Mann–Whitney U test were used appropriately. Spearman’s rank correlation coefficients were used to calculate the correlations between the SMI and clinical variables. For categorical or ordinal variables, logistic regression analysis was performed. Cox regression analysis was performed to estimate the risk of intestinal resection. After univariate analysis, all variables with P values less than 0.15 were considered in subsequent multivariate analyses. Cumulative operation-free survival rate was calculated using the Kaplan–Meier method. The log-rank test was used to determine the statistical difference between groups. *P* values were two-sided, and those less than 0.05 were considered statistically significant.

## Results

The study participants included total 72 IBD patients (CD: 43 cases; UC: 29 cases). The clinical backgrounds of the patients are shown in Table 1. Sarcopenia, defined as an SMI below the cut-off value, was observed in 42% (30/72) of the IBD patients. A poor nutritional status, as assessed by O-PNI and CONUT, was significant in UC patients. Height and serum albumin levels were significantly lower in UC patients than in CD patients. Conversely, age was significantly higher in UC patients than in CD patients.

**Table 1:** Clinical backgrounds and outcomes of the patients.

		Crohn’s disease	Ulcerative colitis	*P* value
Male/female		35/8	18/11	0.121^a^
**Age (years), median (IQR)**		**29.0 (25.0–37.0)**	**39 (28–55)**	**0.015**^**b**^
Body mass index, median (IQR)		19.4 (17.6–21.9)	18.9 (17.3–20.1)	0.424^**b**^
Body weight (kg), median (IQR)		55.2 (51.0–59.5)	51.5 (44.0–57.8)	0.052^**b**^
**Height (cm), median (IQR)**		**170 (166–174)**	**165 (156–169)**	**0.002**^**b**^
Skeletal muscle index (cm^2^/m^2^), median (IQR)		45.1 (40.4–52.5)	40.7 (35.7–47.1)	0.059^**b**^
Sarcopenia/non-sarcopenia		16/27	14/15	0.489^a^
Days of admission, median (IQR)		28.0 (15.5–54.0)	30.0 (25.0–40.0)	0.854^**b**^
Intestinal resection (yes/no)		19/24	6/23	0.071^a^
Disease type	Ileal/colonic/ileocolonic	13/4/26	-	
	Pancolitis / left-sided	-	22/7	
Disease activity	CDAI, median (IQR)	227 (159–298)	-	
	Lichtiger score, median (IQR)	-	12 (10–14)	
Endoscopic activity	SES-CD, median (IQR)	13 (8–16)	-	
	UCEIS, median (IQR)	-	6 (5–7)	
**Onodera’s prognostic nutritional index, median (IQR)**		**37.2 (32.1–42.3)**	**31.2 (27.0–34.7)**	**0.005**^**b**^
**Controlling nutritional status, median (IQR)**		**6 (3–9)**	**8 (6–10)**	**0.020**^**b**^
Subjective global assessment, median (IQR)		2 (2–3)	2 (2–3)	1.000^**b**^
Malnutrition universal screening tool, median (IQR)		2 (1–4)	3 (2–3)	0.166^**b**^
Nutritional risk screening 2002, median (IQR)		3 (2–4)	4 (3–4)	0.218^**b**^
BEE (kcal), median (IQR)		1417 (1331–1527)	1394 (1241–1489)	0.181^**b**^
BEE / BW (kcal/kg), median (IQR)		27.1 (25.3–27.7)	26.6 (24.5–28.0)	0.709^**b**^
REE (kcal), median (IQR)		1339 (1246–1590)	1358 (1275–1486)	0.865^**b**^
REE / BW (kcal/kg), median (IQR)		26.3 (24.5–28.9)	28.0 (24.3–29.7)	0.476^**b**^
Respiratory quotient, median (IQR)		0.80 (0.78–0.85)	0.74 (0.72–0.83)	0.082^**b**^
Hematocrit (%), median (IQR)		35.1 (29.7–39.8)	30.8 (27.8–36.0)	0.059^**b**^
White blood cell count (/μL), median (IQR)		7,500 (5,700–9,850)	9,000 (7,500–12,000)	0.055^**b**^
Total lymphocyte count (/μL), median (IQR)		1,295 (826–1,619)	1,068 (884–1,527)	0.584^**b**^
Neutrophil / Lymphocyte ratio, median (IQR)		4.41 (2.72–8.68)	6.14 (4.54–8.71)	0.054^**b**^
**Albumin (g/dL), median (IQR)**		**3.0 (2.7–3.4)**	**2.6 (2.1–2.9)**	**0.002**^**b**^
Total cholesterol (mg/dL), median (IQR)		130 (116–151)	120 (102–147)	0.502^**b**^
C-reactive protein (mg/dL), median (IQR)		2.82 (0.57–9.49)	4.30 (2.05–8.39)	0.165^**b**^

BW: body weight, CDAI: Crohn’s disease activity index, SES-CD: simple endoscopic score for Crohn’s disease, UCEIS: ulcerative colitis endoscopic index of severity, BEE: basal energy expenditure, REE: resting energy expenditure, IQR: interquartile range. Bold items indicate statistically significant results.

^a^χ2–test

^b^Mann–Whitney U test.

**Fig 1:**
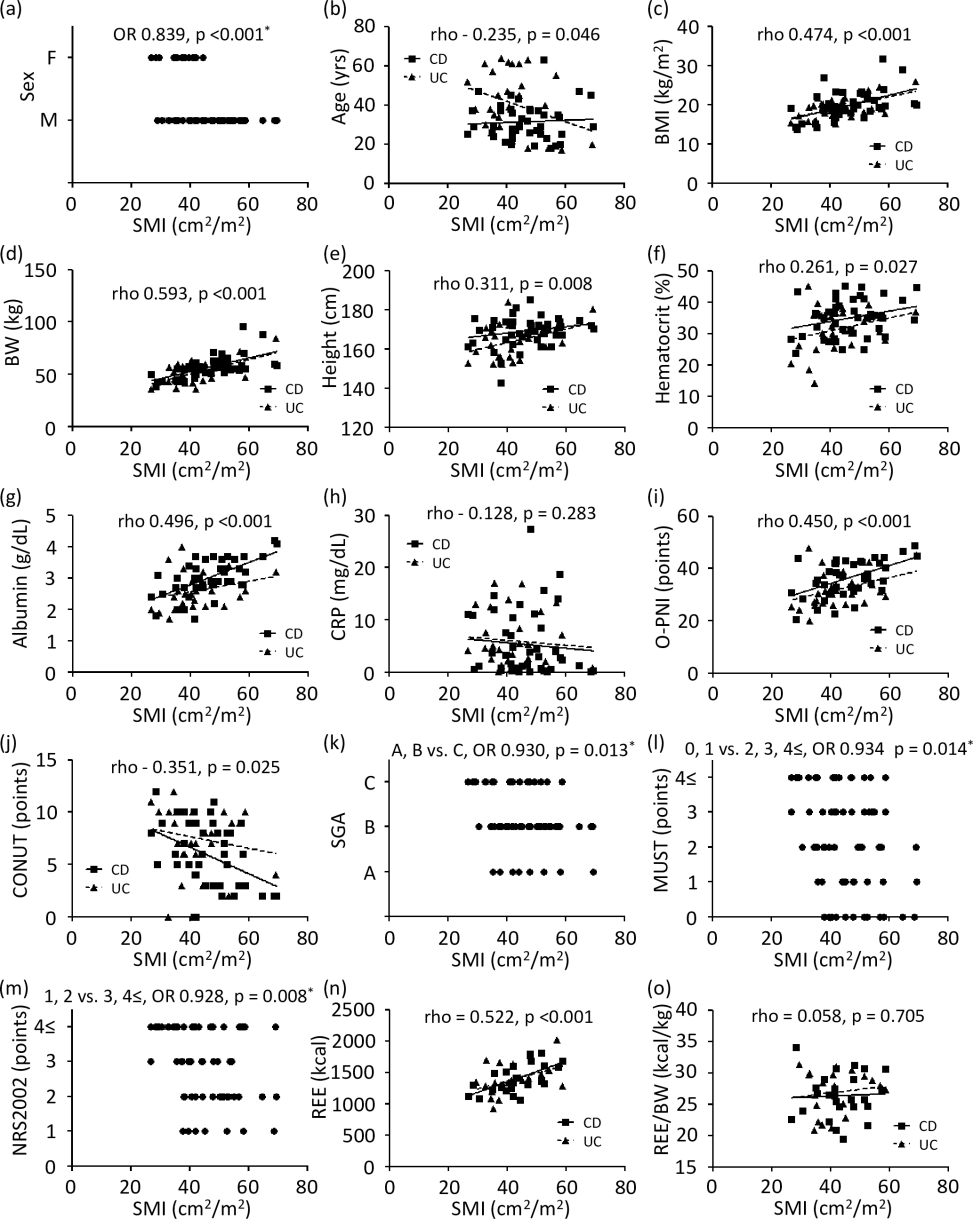
**Dispersion graphs depicting correlations between SMI and sex (a), age (b), BMI (c), BW (d), height (e), hematocrit (f), albumin (g), CRP (h), O-PNI (i), CONUT (j), SGA (k), MUST (l), NRS-2002 (m), REE (n), and REE/BW (o).** *P* values on each graph were calculated for all IBD patients. Rho indicates Spearman’s rank correlation coefficient. *Logistic regression analysis. OR: odds ratio, SMI: skeletal muscle index, BMI: body mass index, BW: body weight, CRP: C-reactive protein, O-PNI: Onodera’s prognostic nutritional index, CONUT: controlling nutritional status, SGA: subjective global assessment, MUST: malnutritional universal screening tool, NRS-2002: nutritional risk screening-2002, REE: resting energy expenditure.

Correlations between the SMI and clinical variables were assessed (Fig 1). In IBD patients, the variables that were significantly correlated with the SMI were sex, age, body mass index, body weight, height, hematocrit, albumin level, O-PNI, CONUT, SGA, MUST, NRS-2002, and REE. To assess the correlation between the nutritional indices and SMI, SGA, MUST, and NRS-2002 were divided into two groups and evaluated by logistic regression analysis (shown in Fig 1). SGA, MUST, and NRS-2002 showed significant relationships with the SMI. Although REE was significantly correlated with the SMI, the significance was diminished as REE was normalized by body weight. Of all variables, the SMI has a strong correlation to sex, body weight, albumin level, and O-PNI.

Factors associated with intestinal resection were assessed by Cox regression analysis (Table 2). Univariate analysis revealed the presence of sarcopenia, disease type (CD or UC), and length of hospital stay as the most significant factors predicting surgery. Multivariate analysis using variables obtained at the time of hospitalization identified the presence of sarcopenia and disease type (CD or UC) as the most significant factors predicting surgery.

**Table 2:** Factors associated with intestinal resection for all IBD patients.

All (n = 60)	Univariate HR (95% CI)	Multivariate HR (95% CI)
Age	0.982 (0.951–1.014), 0.263	-
Sex (Male/Female)	0.890 (0.355–2.233), 0.804	-
Body mass index	1.006 (0.873–1.159), 0.935	-
Skeletal muscle index	0.959 (0.918–1.003), 0.067	-
Sarcopenia/Non-sarcopenia	**0.313 (0.138–0.712), 0.006**	**0.318 (0.126–0.802), 0.015**
Disease (CD/UC)	**0.382 (0.152–0.959), 0.040**	**0.278 (0.108–0.714), 0.007**
O-PNI	0.957 (0.903–1.015), 0.142	-
Hemoglobin	0.970 (0.876–1.076), 0.573	-
Total lymphocyte count	0.999 (0.999–1.001), 0.728	-
Neutrophil lymphocyte count	1.037 (0.967–1.112), 0.302	-
Albumin	0.592 (0.302–1.156), 0.124	0.782 (0.354–1.726), 0.542
C-reactive protein	1.062 (0.994–1.134), 0.073	1.059 (0.985–1.137), 0.119
Length of stay	**1.058 (1.039–1.078), <0.001**	-

Surgery was required 21 for patients (35%). Univariate and multivariate analysis were conducted by Cox regression analysis. Bold numbers indicate statistically significant results. CD: Crohn’s disease, UC: ulcerative colitis, CI: confidence interval

**Fig 2:**
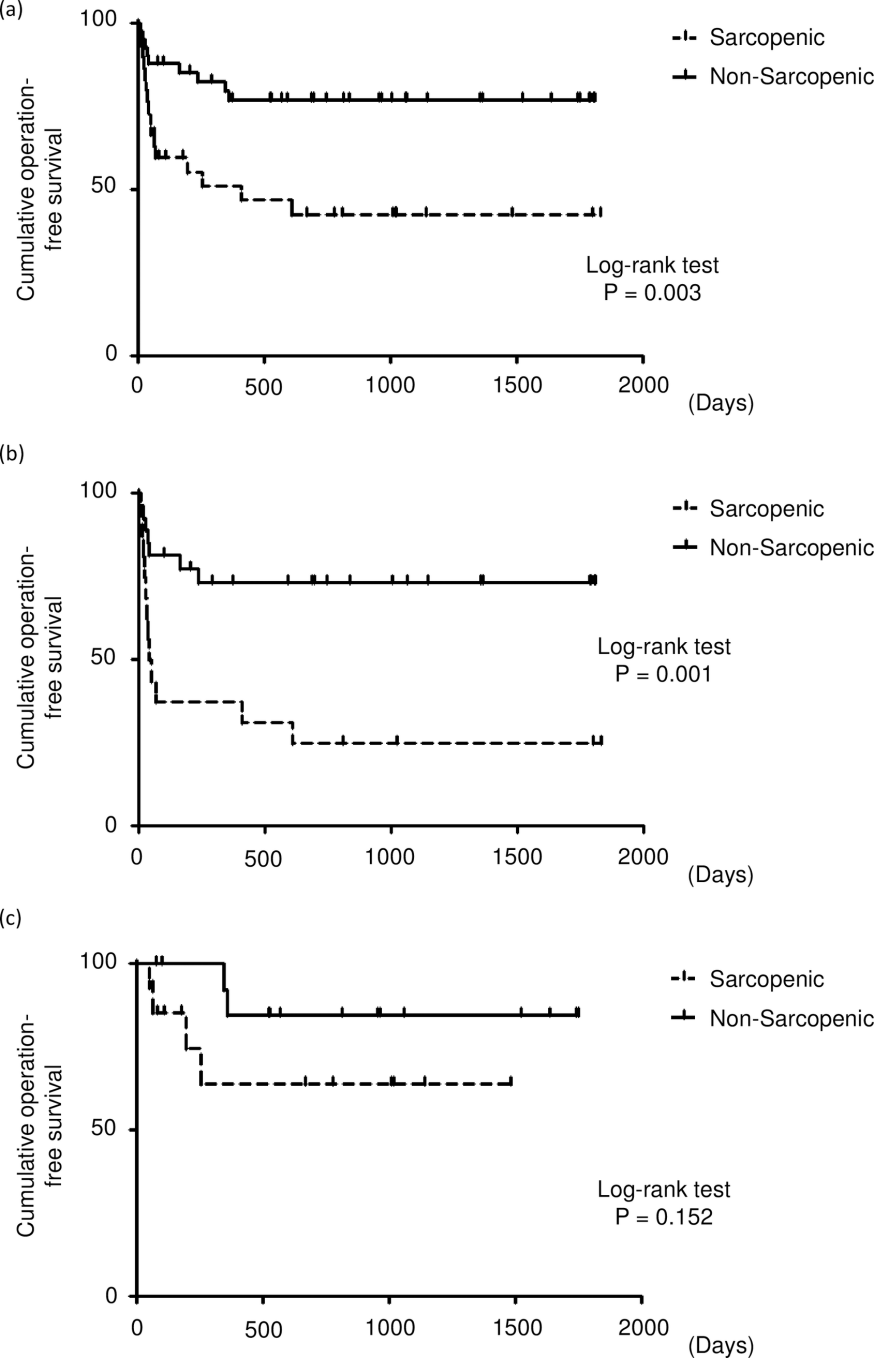
Cumulative operation-free survival rate for all patients (a), patients with Crohn’s disease (b), patients with ulcerative colitis (c).

A Kaplan–Meier analysis was used to calculate the cumulative operation-free survival rate (Fig 2). Among all IBD patients, those with sarcopenia had a significantly lower cumulative operation-free rate than those without. A significant association between intestinal resection and sarcopenia was observed in CD patients. In UC patients, there was no significant difference between patients with and without sarcopenia in the cumulative operation-free survival rate.

**Fig 3:**
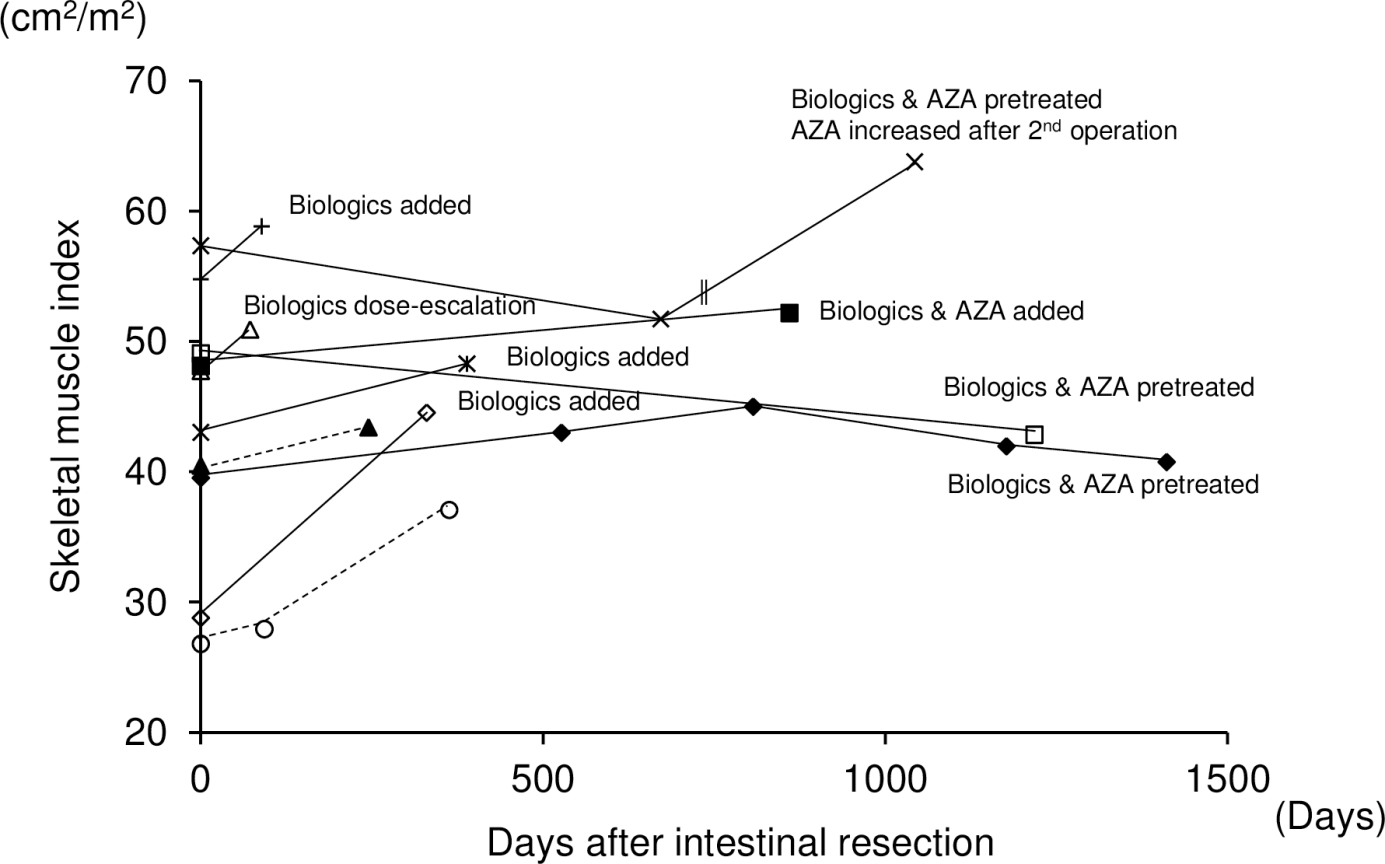
Changes of SMI after intestinal resection. Solid and dashed line indicates patients with CD and UC, respectively. Double vertical lines indicate intestinal resection. AZA: azathioprine.

Post-operative course and the changes of SMI were summarized in Fig 3. Follow-up CT was obtained from eleven patients (CD: 9 cases; UC: 2 cases). The SMI was increased after intestinal resection. However, the recovery of SMI was not observed in patients with CD who had intestinal resection although treated with biologics and azathioprine.

## Discussion

In this study, we enrolled IBD patients who were admitted to our hospital for disease exacerbation, and we demonstrated that a low SMI was a strong predictor of intestinal resection in CD patients. Previous reports have shown that altered body composition or decreased skeletal muscle volume was frequently observed in IBD patients [7–9]. Using dual-energy X-ray absorptiometry, Schneider et al. revealed that 60% of CD patients in clinical remission had sarcopenia [9]. Zhang et al. also reported that 61.4% of CD patients had sarcopenia assessed by cross-sectional images at the L3 level to assess skeletal muscle volume in CD patients with the cut-off value of SMI <55 cm^2^/m^2^ for men and <39 cm^2^/m^2^ for women [11]. Although this percentage is higher than that seen in our results, the frequency of aberrations in IBD patients varies by the population, assessment tools, and cut-off values. Appropriate cut-off values for IBD patients have not yet been determined. Therefore, the study of additional cases is necessary to determine the appropriate cut-off values for the SMI in IBD patients.

The nutritional status of UC patients was poorer than that of CD patients. UC can present as an acute severe extensive disease at the time of exacerbation [40] and can cause a rapid decrease in serum albumin levels. The nutritional indices O-PNI and CONUT showed a significant decrease in UC patients; notably, both indices include measurements of serum albumin level as a component of their respective calculations.

Previous reports demonstrated that skeletal muscle volume is strongly correlated with sex, age, body weight, and serum albumin levels [16–18, 20]. Our results showed no significant relationship between the SMI and serum albumin levels in UC patients. These results suggest that decreases in the skeletal muscle volume occur more slowly than decreases in serum albumin levels during an acute severe extensive disease. Conversely, there is a strong correlation between the SMI and serum albumin level in CD patients. In CD, serum albumin levels are considered to reflect chronic and persistent inflammation or intestinal deformities, such as stenosis and fistula.

Our results revealed a strong association between the SMI and intestinal resection in CD patients. In previous reports, a strong association between sarcopenia and prognosis has been reported in the field of malignant neoplasms and chronic diseases [16–22], most of which present irreversible and progressive clinical courses. Indications for intestinal resection differ between CD and UC. CD requires intestinal resection due to intestinal deformities, such as stenosis and fistula, and UC warrants resection for uncontrollable intestinal inflammation, such as bleeding or perforation. CD is often accompanied by irreversible complications, such as strictures or fistula, as shown by the Lémann score [41]. Therefore, an intestinal deformity may lead to PEM and result in a decreased SMI. In contrast, medical treatment sometimes brings about dramatic outcomes in UC patients. Therefore, the association between the SMI and intestinal resection is considered weak in UC.

Recovery of skeletal muscle volume was reported after induction of infliximab in CD patients [42] or after colectomy in UC patients [43]. We have demonstrated that post-operative changes of the SMI in 11 patients. Although the number of the patients in our cohort is limited, our results confirmed the same trend as the previous reports.

Our study has limitations by its single center and retrospective nature. First of all, the number of patients enrolled in this study is limited, hence the significance of each parameter should be confirmed in the prospective multicenter study. Secondly, there are some missing data in endoscopic assessment and indirect calorimetry. Endoscopic evaluation was not performed in 2 out of 43 CD patients. As for indirect calorimetry, the data was available in 42 out of 72 IBD patients. These parameters are not strongly related to the SMI (data not shown). Therefore, the missing data do not affect the conclusions. Thirdly, this study is observational study. It is necessary to consider whether intervention such as rehabilitation and nutritional therapy is related to prognosis and the SMI in the future study.

In conclusion, L3 skeletal muscle area can serve as a prognostic factor for intestinal resection in IBD patients, especially in those with CD. These results may reflect the fact that CD presents as a gastrointestinal disease arising from intestinal deformities.

## Supporting information

S1 File The raw data of all patients. (XLSX)
